# Canadian genetic healthcare professionals’ attitudes towards discussing private pay options with patients

**DOI:** 10.1002/mgg3.572

**Published:** 2019-02-02

**Authors:** Vanessa Di Gioacchino, Sylvie Langlois, Alison M. Elliott

**Affiliations:** ^1^ Department of Medical Genetics University of British Columbia Vancouver British Columbia Canada; ^2^ BC Children’s Hospital Research Institute Vancouver British Columbia Canada; ^3^ Women’s Hospital Research Institute Vancouver British Columbia Canada

**Keywords:** genetic counseling, genomic testing, policy, private pay

## Abstract

**Background:**

Just as there is inconsistency with respect to coverage of genomic testing with insurance carriers, there is interprovincial discrepancy in Canada. Consequently, the option of private pay (e.g., self pay) arises, which can lead to inequities in access, particularly when patients may not be aware of this option. There are currently no published data regarding how the Canadian genetics community handles discussions of private pay options with patients. The purpose of this study was to assess the attitudes of genetic healthcare professionals (GHPs: medical geneticists, genetic counselors, and genetic nurses) practicing in Canada toward these discussions.

**Methods:**

An online survey was distributed to members of the Canadian College of Medical Geneticists and the Canadian Association of Genetic Counsellors to assess frequencies, rationale, and ethical considerations regarding these conversations. Quantitative data were analyzed using descriptive statistics.

**Results:**

Of 144 respondents, 95% reported discussing private pay and 65% reported working in a clinic without a policy on this issue. There were geographic and practice‐specific differences. The most common circumstance for these discussions was when a test was clinically indicated (e.g., but funding was denied) followed by when the patient initiated the conversation. The most frequently discussed tests included: multi‐gene panels (73% of respondents), noninvasive prenatal testing (62%), and pre‐implantation genetic diagnosis (58%). Although 65% felt it was ethical to discuss private pay, 35% indicated it was “sometimes” ethical.

**Conclusion:**

With the increasing availability of genomic technologies, these findings inform how we practice and demonstrate the need for policy in this area.

## 1 INTRODUCTION

In Canada, healthcare services are publicly funded and the coverage of “medically necessary” services is mandated by the Canada Health Act (CHA) (Government of Canada, 1985). However, healthcare falls under provincial jurisdiction and the CHA leaves the interpretation of “medically necessary” to each provincial or territorial government (Stradiotto, 2007), leading to interprovincial differences in covered services. Further, continuous advances in technology and medicine offer innovative, yet expensive, diagnostic and therapeutic options, which the government may not cover despite being clinically valuable (Stradiotto, 2007). As a result, difficult resource allocation decisions need to be made. Medical genetics is not immune to the effects of these decisions and, as a result, some clinically indicated genetic testing may not be covered (Rogowski, Grosse, Schmidtke, & Marckmann, 2014). Tests may be clinically indicated for a variety of reasons including: the patient's clinical presentation, medical history or family history. Patients may wish to pursue genetic testing for psychological well‐being (e.g., due to anxiety). Patients may have the option to pay privately for these tests, either themselves or through extended insurance plans; however, not all patients may be aware of this. For the purpose of this study, “private pay” refers to self pay (the patient paying out of pocket) either directly or through an extended private insurance plan.

There are several reasons why genetic healthcare professionals (GHPs, e.g., medical geneticists, genetic counselors, and genetic nurses) may or may not discuss private pay options with their patients. These can include, but are not limited to, whether the GHP thinks the patient can afford to pay for the test or whether they believe that this option contributes to a two‐tier health system (Drazba, Kelley, & Hershberger, 2014; MacDonald, 2002). Anecdotally, practice varies at the level of the individual GHP. However, there are currently no published data regarding how this issue is handled by the Canadian genetics community. There are ethical implications as this may lead to inequitable access to health care across the country, across a given province, or even within a clinic. However, GHPs in Canada do not currently have national practice guidelines available to them to help navigate these complex situations.

The purpose of this study was to describe the frequency at which private pay options are discussed with patients for whom genetic testing is clinically indicated (e.g., as per clinical guidelines), and under which circumstances. This study provides insight into the decision‐making of GHPs with respect to discussing private pay options with patients, including the reasoning behind their decisions and any discordance that may exist with respect to the rationale regarding the dialog. Identifying these underlying issues can foster an open discussion on the issue of access to genetic services. It could lead to policy development addressing these discrepancies so that Canadians may be equally informed of available options, which may influence their own decision‐making when considering genetic testing.

## 2 METHODS

### 2.1 Ethical Compliance

Ethics approval for this study was obtained through the Children's and Women's Research Ethics Board and University of British Columbia in Vancouver, British Columbia (H17‐00225).

### 2.2 Study design & survey development

The authors developed the questionnaire and received feedback on a draft version from five GHPs. This feedback was incorporated into the final version, which consisted of 15 multiple choice and free response questions intended to gather information about participant demographics and frequencies, rationale, and ethical considerations around private pay discussions (see Questionnaire S1). The authors estimated that it would take each participant approximately 10–15 min to complete the questionnaire.

The questionnaire utilized the phrase “private pay” to refer to any genetic testing that was indicated for the patient based on the GHP's clinical judgement and paid for by an entity other than the provincial government (typically the patients themselves but, rarely, could include private insurance companies); it does not refer to direct‐to‐consumer genetic testing.

### 2.3 Survey administration & setting

The online survey was administered using the REDCap tool (Harris et al., 2009), available through the British Columbia Children's Hospital Research Institute (BCCHR). A link to the online questionnaire was circulated via the e‐mail distribution lists of the Canadian College of Medical Geneticists (CCMG, https://www.ccmg-ccgm.org) and the Canadian Association of Genetic Counsellors (CAGC, https://www.cagc-accg.ca). The survey was open for participation for 4 months.

All English‐speaking or bilingual (French and English) genetic counselors, medical geneticists, and other genetic healthcare providers actively practicing in Canada were eligible to participate. This was expected to encompass “regular” members of CCMG and “full” members of CAGC (those who have completed training and/or certification and are practising; these categories exclude trainees and emeritus members of both professions): there were 244 regular members of CCMG at the time of survey circulation and 306 full members of CAGC. Only responses that were complete and where participants clicked “submit” to provide implied consent were included.

### 2.4 Data analysis & primary measurements

Descriptive statistics were utilized to evaluate participant demographics, the frequency of private pay discussions by GHPs, genetics clinic policies on private pay, circumstances that influence private pay discussions, types of private pay genetic testing that are discussed Canada‐wide, and ethical considerations, as well as to identify any discrepancies that emerged amongst GHPs in different regions (British Columbia, the Prairie provinces (Alberta, Saskatchewan and Manitoba), Ontario, Quebec, and the Maritime provinces (Newfoundland and Labrador, Nova Scotia, Prince Edward Island and New Brunswick)), subspecialties of practice, or type of GHP. One participant reported being both a genetic counselor and a medical geneticist; this was reflected in the demographic table but this participant was considered as only a medical geneticist for the remainder of the analyses. One participant reported practising in all provinces and territories; this participant was excluded from analyses by geographical region. Some participants’ responses to the “other” option for some questions echoed options that were available within that question, so the responses of 10 participants were modified prior to the final analysis to reflect this (i.e., if a participant selected “other” for the demographic question regarding Area of Practice and wrote “pediatric cancer,” the response was modified to remove “other” and include “pediatric” and “cancer”)

Due to some “check all that apply” questions, some questions received more responses than the number of participants. Percentages are calculated as percent of participants included within each category rather than the percent of responses. Further, some participants’ responses were included in multiple categories within one comparison (i.e., if they reported practicing in both cancer and prenatal genetics, they were included in both categories in the analyses).

Qualitative data were not formally analyzed, but were reviewed to identify common themes in order to provide context for quantitative findings.

## 3 RESULTS

### 3.1 Survey response

In total, 153 responses were received for a response rate of 28%. Nine responses were excluded: four respondents indicated they were not currently practicing in Canada, four had incomplete questionnaires, and one was identified as a duplicate, leaving 144 responses (26%; an estimated 10% participation rate from CCMG and 39% from CAGC). Demographic features are summarized in Table 1. The majority of respondents were female, English‐speaking, and practicing as genetic counselors.

**Table 1:** Demographics of survey participants

	Genetic counselors & nurses (%) *n* = 120	Clinical geneticists (%) *n* = 25	Total (%) *n* = 144
Gender			
Male	5 (4)	7 (28)	12 (8)
Female	115 (96)	18 (72)	132 (92)
Language			
English	94 (78)	20 (80)	114 (79)
French	2 (2)	2 (8)	4 (3)
Both	23 (19)	3 (12)	26 (18)
Professional designation^a^			
Clinical Geneticist (MD)	1 (1)	25 (100)	25 (17)
Genetic Counselor	118 (98)	1 (4)	119 (83)
Nurse	1 (1)	—	1 (1)
PhD Geneticist	—	2 (8)	2 (1)
Specialization^a^			
Adult	59 (49)	18 (72)	76 (53)
Cancer	60 (50)	10 (40)	69 (48)
Lab	10 (8)	3 (12)	13 (9)
Pediatric	44 (37)	18 (72)	62 (43)
Prenatal	51 (43)	11 (44)	62 (43)
Subspecialty or research^b^	52 (44)	21 (84)	73 (51)
Region of practice^a^			
British Columbia (BC)	28 (24)	4 (16)	32 (22)
Prairies^c^	13 (11)	6 (24)	19 (13)
Ontario (ON)	50 (41)	11 (44)	60 (42)
Quebec (QC)	21 (18)	3 (12)	24 (17)
Maritimes^d^	14 (12)	1 (4)	15 (10)
Territories^e^	1 (1)	—	1 (1)
Years of practice			
0–2	21 (18)	4 (16)	25 (17)
3–5	24 (20)	4 (16)	28 (19)
6–10	20 (16)	5 (20)	24 (17)
11–2	36 (30)	4 (16)	40 (28)
>20	19 (16)	8 (32)	27 (19)

a Category totals may be discordant due to “check all that apply” questions; percentages are calculated as percent of participants rather than percent of responses.

b Subspecialties include: cardiac, fertility/assisted reproductive technologies, metabolic, neurology, newborn screening, psychiatric, and ocular.

c Alberta, Saskatchewan, and Manitoba.

d Newfoundland and Labrador, Nova Scotia, Prince Edward Island, and New Brunswick.

e Yukon Territory, Northwest Territories, and Nunavut.

### 3.2 Discussion of private pay testing

In total, 95% of participants reported discussing private pay options with patients (Table 2); 100% of participants from British Columbia and the Prairie provinces reported they discuss private pay compared to 86% of participants from the Maritime provinces.

**Table 2:** Participants’ responses as to whether they discuss private pay options for genetic testing with patients

	GC (%) *n* = 119	MD (%) *n* = 25	Cancer (%) *n* = 69	Prenatal (%) *n* = 62	Other (%) *n* = 110	BC (%) *n* = 31	Prairies (%) *n* = 18	ON (%) *n* = 59	QC (%) *n* = 23	Maritimes (%) *n* = 14	Total (%) *n* = 144
Yes	113 (95)	24 (96)	68 (99)	61 (98)	104 (95)	31 (100)	18 (100)	55 (93)	21 (91)	12 (86)	137 (95)
No	6 (5)	1 (4)	1 (1)	1 (2)	6 (5)	—	—	4 (7)	2 (9)	2 (14)	7 (5)

Note

GC, genetic counselor; MD, medical doctor/geneticist; BC, British Columbia; Prairies, Alberta, Saskatchewan, and Manitoba; ON, Ontario; QC, Quebec; Maritimes, Newfoundland and Labrador, Nova Scotia, Prince Edward Island, and New Brunswick.

### 3.3 Clinic policies

Twenty‐two percent of participants reported working in a clinic that has a policy on discussing private pay options (Table S1). However, most participants described the policy as “informal or unwritten” (data not shown). Of participants who work in a clinic with a policy, most (78%) described it as permissive or encouraging of private pay discussions and the remainder (22%) described it as discouraging or preventative. The majority of participants (65%) reported that their clinic or group does not have a policy on this issue and 13% were unaware if there was such a policy.

### 3.4 Circumstances and types

The most common circumstances under which GHPs would discuss private pay genetic testing (Table S2) were when a clinically appropriate test was not funded, including when a specific funding request was denied (85%). The next most common reason for discussing private pay testing was when the patients initiated the conversation by asking whether they could pay for the test themselves or whether there were any other options available (35%). Two percent of participants reported basing their decisions to discuss these options on their perception of whether a patient could afford to pay for the test.

The three most common types of genetic tests discussed as private pay options (Table S2) were multi‐gene sequencing panels (“panels”), noninvasive prenatal testing (NIPT), and pre‐implantation genetic diagnosis (PGD). Panels were reportedly discussed by 73% of all participants, most frequently in British Columbia (90%) and the Prairies (89%) and least frequently in the Maritimes (50%). Every participant practising solely in cancer genetics (100%) reported discussing panels as private pay options (Table S3). With respect to NIPT, 62% of all participants reported discussing this as a private pay option with patients; and included 90% of participants whose practice includes prenatal genetics and 33% of participants from the Prairies. Thirdly, 58% of participants reported that they would discuss PGD as a private pay option with their patients; more specifically, 71% of participants in the Maritimes and 48% of participants from British Columbia. The three types of genetic tests least frequently reported to be discussed as private pay options were first trimester screening (nine percent), genome‐wide sequencing (10%), and “others” (10%), such as expanded carrier screening.

### 3.5 Influencing factors

The majority (83%) of participants identified one of four factors as the most influential in their decision to discuss private pay (Table S4): the impact on medical management (26%), the patient's risk (21%), the patient's anxiety level (19%), and ineligibility for funding (17%). Two other factors were selected by many participants as influential to the decision but were not widely selected as the most influential factor: the impact on family planning (influential for 67%, most influential for four percent) and the psychological impact of having results (influential for 49%, most influential for none).

### 3.6 Ethical considerations

When deciding whether discussing private pay options within a publicly funded healthcare system was ethical (Table S5), 65% of participants responded “yes,” 34% “sometimes,” and one percent “no.” The main ethical principle that drove that decision was patient autonomy to make his or her own decision after knowing all the available options (56%), rather than justice, beneficence, or nonmaleficence.

## 4 DISCUSSION

This is the first study to report on the practice of Canadian GHPs with respect to discussion of private pay genetic testing options. Our findings show that, while most GHPs report discussing these options with patients, there is inconsistency amongst practices, including the types of testing that may be discussed, the circumstances under which the option may be presented, the factors that may influence their decisions and whether policies are in place at the clinics where they practice. The manner in which private pay options are discussed may impact patients’ perceptions of the options and the decisions they ultimately make about whether to pay privately for genetic testing. Consequently, inconsistencies amongst GHPs may lead to inequities regarding patient care and different decision‐making patterns of patients. These results emphasize the need for uniformity of practice amongst Canadians GHPs.

### 4.1 Types of genetic testing discussed

When discussing private pay genetic tests, multi‐gene panels were reported to be the most commonly discussed type of genetic test. Panels may be ordered for a variety of indications, including hearing loss, cardiac conditions, and cancer. While our survey did not specifically ask about the indications for which GHPs were discussing panels, 100% of participants who practice solely in cancer genetics reported discussing private pay panels with patients. Panels are a common test in cancer genetics (Pederson et al., 2018) and many of the survey participants indicated that they commonly present private pay options in the context of cancer genetic counseling, usually for “unaffected individuals with a family history suggestive of hereditary cancer for which they themselves do not meet criteria for testing and more appropriate individuals for testing are not able to be tested.” This appears to be consistent with practice guidelines for cancer genetics, which indicate that testing is best started with an affected family member as testing unaffected individuals is less informative (Berliner, Fay, Cummings, Burnett, & Tillmanns, 2013; Daly et al., 2018; Provenzale et al., 2017).

Noninvasive prenatal testing was the second most frequent private pay genetic test discussed. Canadian, American, and Australian practice guidelines recommend discussing NIPT with pregnant women in varying situations (Audibert et al., 2018; Gregg et al., 2016; O'Leary et al., 2016) but not all Canadian women who meet guideline criteria may be eligible for funded NIPT in their home province (Gekas et al., 2016; Vanstone, King, de Vrijer, & Nisker, 2014), which may lead to some GHPs feeling compelled to discuss the option for the patient to pay privately for the test. This was illustrated by one participant writing, “per SOGC guidelines, we present NIPT as an option whenever amniocentesis is being discussed (exceptions: if there are multiple congenital anomalies where microarray on amnio[centesis] may have a greater yield…).” Further, physicians are required to disclose all of the risks, benefits, and alternatives to tests or procedures being discussed (Toews & Caulfield, 2018), which may also lead a GHP to discuss NIPT despite it being unfunded in order to avoid a medico‐legal liability issue. In addition, although NIPT can only be ordered through a physician, it is heavily marketed directly to patients, which may have contributed to a rapid increase in patient inquiries about this test (Gekas et al., 2016).

The third most commonly discussed genetic test was pre‐implantation genetic diagnosis (PGD). To our knowledge, this test is not currently funded anywhere in Canada, although some provinces may have funding or tax deductions for in vitro fertilization (IVF) cycles. In general, guidelines on reproductive genetic testing require that the condition in question be severe enough to warrant the testing, but the level of risk required is not defined (Knoppers & Isasi, 2004). This ambiguity makes determining whether PGD is an available option for a given patient more challenging and can lead to inconsistency in patients being offered this service.

### 4.2 Geographic differences

Not all participants reported discussing private pay testing with their patients. The proportion of GHPs who reported not discussing private pay options appeared to be relatively higher in the Maritime provinces at 14%, whereas no GHPs from British Columbia or the Prairie provinces reported that they do not discuss private pay. It cannot be determined from this study whether the reason for this discordance is related to clinic policies, interprovincial differences in funding, or other factors. However, this further demonstrates the inconsistency in practice.

### 4.3 Inconsistency of access and need for policy

There are many conflicting reasons why a GHP may choose to discuss private pay options. From the open responses written by survey participants, these can include but are not limited to: “it is unfair that the patient has to pay”, “sometimes it is the only option for getting the family what they need/desire”, “I am the person responsible for making sure the patient is informed”, and “I am reluctant to contribute to utilizing government resources (my time) to coordinate private pay testing.” GHPs may weigh pros and cons differently impacting whether to discuss private pay options. Consequently, patients may receive discrepant information about their options, resulting in unequal access to healthcare options. In addition, private pay testing may only be a feasible option for patients who can afford to pay for the tests (de Jong & de Wert, 2015), further contributing to inequality in healthcare access.

The demand for genetic counseling that comes with an increase in genetic testing cannot be met by the current number of GHPs in Canada (Gekas et al., 2016). This illustrates the need for a policy that determines the best practice within the context of the Canadian healthcare system and helps guide clinics and GHPs on this issue so that genetics programs can appropriately allocate time and resources accordingly. Sixty‐five percent of GHPs reported that their group does not have a policy on discussing private pay testing with patients. Further, a national policy or practice guideline does not currently exist. The Canadian College of Medical Geneticists has issued a position statement regarding direct‐to‐consumer genetic testing (Nelson et al., 2012), which is a different issue from clinically indicated genetic testing that is paid for by the patient because it is not funded by the provincial health services plan. Policy on this issue leads to consistency in the care patients receive and protects providers from liability issues (Boycott et al., 2015; Toews & Caulfield, 2018).

### 4.4 Study limitations

Due to a low‐to‐average response rate (Nulty, 2008) from the size‐limited population of Canadian GHPs (and a particularly low response rate from CCMG members), this study had a small sample size. As a result, tests for statistical significance could not be performed on this data and descriptive statistics were used. While the geographic (Table S6) and gender distributions of participants appeared to reflect those of full CAGC membership within Canada, it is possible that some GHPs without an interest in discussing private pay genetic testing may have chosen not to participate in this study. Consequently, there may ascertainment bias due to the sample of GHPs who chose to participate.

### 4.5 Conclusion and future directions

The purpose of this study was not to discuss the potential implications of specific genetic test results that may arise from private pay testing, such as incidental findings, variants of uncertain significance, or cascade testing. The purpose was to describe current practice in Canada. Our findings show that there is inconsistency of practice on the topic of private pay genetic testing amongst Canadian GHPs. However, this problem could be resolved by the development of practice guidelines to lessen the decision‐making burden for individual GHPs (Boycott et al., 2015). Policy development would also guide training for GHPs (genetic counseling students, medical genetics residents, and fellows), instilling consistency of practice. This study focused on the practice of GHPs, but other medical professionals also discuss genetic testing with their patients, including family physicians, obstetricians, midwives, and oncologists, whose practice on this issue may be even more varied and have significant implications for patient care and provider liability. Policy on this topic would provide support and guidance to all providers.

While willingness to pay was not the focus of our study, it is worth noting that patients in the United States and Australia have been shown to be willing to pay for genetic testing that is not covered by their insurance plans (Georgiou et al., 2016; Lin, Yeh, & Neumann, 2017; Marshall et al., 2016) and Canadians have been shown to be willing to pay for other healthcare services (Guimarães et al., 2009). However, if patients are not aware of this option, their willingness to pay becomes irrelevant. A complementary study could be executed to assess patient preferences for being offered private pay options by their healthcare providers. This information could be incorporated into policy development on this issue.

## CONFLICT OF INTEREST

None declared.
