# Supplementary material for: Canadian genetic healthcare professionals’ attitudes towards discussing private pay options with patients
Source: Mol Genet Genomic Med. 2019 Feb 2;7(4):e00572. doi: 10.1002/mgg3.572 (PMC6465662; doi:10.1002/mgg3.572)
Supplement: Supplementary file 1 [file MGG3-7-na-s001.docx]

**Supplementary Table 1:** Existence and type of clinic policies on private pay discussions

|  | GC (%)  n=119 | MD (%)  n=25 | BC (%)  n=31 | Prairies (%)  n=18 | ON (%)  n=59 | QC (%)  n=23 | Maritimes (%)  n=14 | Total (%)  n=144 |
| --- | --- | --- | --- | --- | --- | --- | --- | --- |
| Does your clinic have a policy? | | | | | | | | |
| Yes, discourages | 5 (4) | 2 (8) | - | 3 (17) | 4 (7) | - | - | 7 (5) |
| Yes, allows | 20 (17) | 5 (20) | 8 (26) | 7 (29) | 10 (17) | - | - | 25 (17) |
| No | 81 (68) | 13 (52) | 18 (58) | 7 (39) | 41 (69) | 17 (74) | 12 (86) | 94 (65) |
| I don’t know | 13 (11) | 5 (20) | 5 (16) | 1 (6) | 4 (7) | 6 (26) | 2 (14) | 18 (13) |
| If “discourages”, do you agree with it? | | | | | | | | |
| Yes | 3 (60) | 1 (50) | - | 2 (67) | 2 (50) | - | - | 4 (57) |
| No | 2 (40) | 1 (50) | - | 1 (33) | 2 (50) | - | - | 3 (43) |
| If “allows”, do you agree with it? | | | | | | | | |
| Yes | 17 (85) | 5 (100) | 7 (88) | 5 (71) | 10 (100) | - | - | 22 (88) |
| No | 3 (15) | - | 1 (13) | 2 (29) | - | - | - | 3 (12) |
