# Supplementary material for: Canadian genetic healthcare professionals’ attitudes towards discussing private pay options with patients
Source: Mol Genet Genomic Med. 2019 Feb 2;7(4):e00572. doi: 10.1002/mgg3.572 (PMC6465662; doi:10.1002/mgg3.572)
Supplement: Supplementary file 2 [file MGG3-7-na-s002.docx]

**Supplementary Table 2:** Circumstances under which private pay is discussed and types of private pay genetic testing that are discussed

|  | GC (%)  n=119 | MD (%)  n=25 | Cancer (%)  n=69 | Prenatal (%)  n=62 | Other (%)  n=110 | BC (%)  n=31 | Prairies (%)  n=18 | ON (%)  n=59 | QC (%)  n=23 | Maritimes (%)  n=14 | Total (%)  n=144 |
| --- | --- | --- | --- | --- | --- | --- | --- | --- | --- | --- | --- |
| Under which circumstances?^a^ | | | | | | | | | | | |
| Whenever it is an option | 28 (24) | 3 (12) | 17 (25) | 15 (24) | 24 (22) | 13 (42) | 1  (6) | 14 (24) | 3 (13) | - | 31 (22) |
| When not automatically funded or funding request is denied | 103 (87) | 20 (80) | 54 (78) | 55 (89) | 95 (86) | 28 (90) | 18 (100) | 46 (78) | 19 (83) | 13 (93) | 122 (85) |
| When I think patient can afford to pay | 3 (3) | - | 2 (3) | 1 (2) | 3 (3) | - | - | 2 (3) | - | 1 (7) | 3 (2) |
| When a patient brings it up first | 46 (39) | 5 (20) | 35 (51) | 24 (39) | 39 (35) | 5 (16) | 6 (33) | 28 (47) | 7 (30) | 5 (36) | 51 (35) |
| Specific circumstances or diagnoses^b^ | 19 (16) | 5 (20) | 14 (20) | 13 (21) | 17 (15) | - | 4 (22) | 10 (17) | 9 (39) | 1 (7) | 24 (17) |
| Which types would you discuss?^a^ | | | | | | | | | | | |
| Non-invasive prenatal testing (NIPT) | 76 (64) | 13 (52) | 34 (49) | 56 (90) | 76 (69) | 20 (65) | 6 (33) | 42 (71) | 10 (43) | 12 (86) | 89 (62) |
| First Trimester Screening (FTS) | 12 (10) | 1 (4) | 2 (3) | 6 (10) | 12 (11) | 6 (19) | - | 5 (8) | 3 (13) | 1 (7) | 13 (9) |
| Single gene sequencing | 48 (40) | 12 (48) | 22 (32) | 25 (40) | 50 (45) | 18 (58) | 9 (50) | 22 (37) | 8 (35) | 4 (29) | 60 (42) |
| Multi-gene panel sequencing | 87 (73) | 18 (72) | 58 (84) | 45 (73) | 76 (69) | 28 (90) | 16 (89) | 40 (68) | 14 (61) | 7 (50) | 105 (73) |
| Whole exome sequencing | 36 (30) | 12 (48) | 13 (19) | 23 (37) | 42 (38) | 19 (61) | 8 (44) | 10 (17) | 3 (13) | 8 (57) | 48 (33) |
| Whole genome sequencing | 12 (10) | 3 (12) | 6 (9) | 6 (10) | 14 (13) | 9 (29) | - | 3 (5) | 1 (4) | 2 (14) | 15 (10) |
| Pre-implantation genetic screening (PGS/CCS) | 32 (27) | 4 (16) | 17 (25) | 20 (32) | 34 (31) | 9 (29) | 1 (6) | 20 (34) | 2 (9) | 5 (36) | 36 (25) |
| Pre-implantation genetic diagnosis (PGD) | 70 (59) | 14 (56) | 39 (57) | 40 (65) | 73 (66) | 15 (48) | 12 (67) | 36 (61) | 12 (52) | 10 (71) | 84 (58) |
| PGD/PGS/CCS only if already considering in vitro fertilization (IVF) | 22 (18) | 4 (16) | 13 (19) | 16 (26) | 17 (15) | 6 (19) | 3 (17) | 14 (24) | 3 (13) | 1 (7) | 26 (18) |
| Other^c^ | 13 (11) | 1 (4) | 6 (9) | 6 (10) | 13 (12) | 1 (3) | - | 10 (17) | 2 (9) | - | 14 (10) |

^a^ Category totals may be discordant due to “check all that apply” questions; percentages are calculated as percent of participants rather than percent of total responses.

^b^ An open-response field allowed participants who selected “specific circumstances or diagnoses” to describe those circumstances; responses included: when PGD could be an option for a couple, when the best-testable in the family is unavailable, when a patient insists on testing or is angry that the testing is not funded, when the couple is consanguineous, when the patient is a non-resident/not yet covered by the provincial healthcare plan, when the condition in question is deemed more serious by the GHP, when practice guidelines recommend discussion of a test that is not funded (i.e. NIPT), and when the patient wants a quicker turn-around time.

^c^ An open-response field allowed participants who selected “other” to name other types of genetic tests; responses included carrier testing, expanded carrier screening, microarray, pharmacogenetic testing, karyotype, and tumour testing.
