# Supplementary material for: Canadian genetic healthcare professionals’ attitudes towards discussing private pay options with patients
Source: Mol Genet Genomic Med. 2019 Feb 2;7(4):e00572. doi: 10.1002/mgg3.572 (PMC6465662; doi:10.1002/mgg3.572)
Supplement: Supplementary file 3 [file MGG3-7-na-s003.docx]

**Supplementary Table 3:** Types of genetic testing that are discussed as private pay options as compared between GHPs practicing solely in cancer genetics and all other GHPs.

|  | Cancer Only (%) n=23 | All Others (%) n=121 | Total (%)  n=144 |
| --- | --- | --- | --- |
| Which types would you discuss?^a^ | | | |
| Non-invasive prenatal testing (NIPT) | 3 (13) | 86 (71) | 89 (62) |
| First Trimester Screening (FTS) | 1 (4) | 12 (10) | 13 (9) |
| Single gene sequencing | 7 (30) | 53 (44) | 60 (42) |
| Multi-gene panel sequencing | 23 (100) | 82 (68) | 105 (73) |
| Whole exome sequencing | 2 (9) | 46 (38) | 48 (33) |
| Whole genome sequencing | 1 (4) | 14 (12) | 15 (10) |
| Pre-implantation genetic screening (PGS/CCS) | 1 (4) | 35 (29) | 36 (25) |
| Pre-implantation genetic diagnosis (PGD) | 7 (30) | 77 (64) | 84 (58) |
| PGD/PGS/CCS only if already considering in vitro fertilization (IVF) | 4 (17) | 22 (18) | 26 (18) |
| Other^b^ | 1 (4) | 13 (11) | 14 (10) |

^a^ Category totals may be discordant due to “check all that apply” questions; percentages are calculated as percent of participants rather than percent of total responses.

^b^ An open-response field allowed participants who selected “other” to name other types of genetic tests; responses included carrier testing, expanded carrier screening, microarray, pharmacogenetic testing, karyotype, and tumour testing.
