# Supplementary material for: Canadian genetic healthcare professionals’ attitudes towards discussing private pay options with patients
Source: Mol Genet Genomic Med. 2019 Feb 2;7(4):e00572. doi: 10.1002/mgg3.572 (PMC6465662; doi:10.1002/mgg3.572)
Supplement: Supplementary file 4 [file MGG3-7-na-s004.docx]

**Supplementary Table 4:** Factors influencing whether a GHP discusses private pay testing options with patients. “All” represents all factors that would influence the participants’ decisions; “most” refers to the single most influential factor for each participant

|  | GC (%)  n=119 | | MD (%)  n=25 | | BC (%)  n=31 | | Prairies (%) n=18 | | ON (%)  n=59 | | QC (%)  n=23 | | Maritimes (%) n=14 | | Total (%)  n=144 | |
| --- | --- | --- | --- | --- | --- | --- | --- | --- | --- | --- | --- | --- | --- | --- | --- | --- |
|  | All^a^ | Most | All^a^ | Most | All^a^ | Most | All^a^ | Most | All^a^ | Most | All^a^ | Most | All^a^ | Most | All^a^ | Most |
| Cost of test | 29 (24) | 2 (2) | 9 (36) | 1 (4) | 12 (39) | 1 (3) | 5 (28) | 1 (6) | 13 (22) | - | 8 (35) | 1 (4) | 1 (7) | - | 38 (26) | 3 (2) |
| Patient’s perceived income | 9 (8) | - | 3 (12) | - | 4 (13) | - | 1  (6) | - | 5 (8) | - | - | - | 2 (14) | - | 12 (8) | - |
| Patient’s perceived understanding | 25 (21) | 3 (3) | 5 (20) | - | 7 (23) | - | 3 (17) | - | 14 (24) | 2 (3) | 4 (17) | - | 3 (21) | - | 30 (21) | 3 (2) |
| Patient’s risk | 94 (79) | 27 (23) | 16 (64) | 3 (12) | 27 (87) | 7 (23) | 10 (56) | 3 (17) | 42 (71) | 13 (22) | 21 (91) | 6 (26) | 11 (79) | 2 (14) | 110 (76) | 30 (21) |
| Patient’s anxiety | 79 (66) | 24 (20) | 14 (56) | 3 (12) | 23 (74) | 5 (16) | 9 (50) | 1 (6) | 36 (61) | 13 (22) | 15 (65) | 3 (13) | 10 (71) | 5 (36) | 93 (65) | 27 (19) |
| Ineligible for funding | 77 (65) | 18 (15) | 21 (84) | 6 (24) | 27 (87) | 7 (23) | 14 (78) | 6 (33) | 39 (66) | 6 (10) | 11 (48) | 4 (17) | 8 (57) | 1 (7) | 98 (68) | 24 (17) |
| Two-tier health system | 13 (11) | 2 (2) | 1 (4) | - | 2 (6) | - | 3 (17) | 1 (6) | 4 (7) | 1 (2) | 4 (17) | - | 2 (14) | - | 14 (10) | 2 (1) |
| Medicolegal issues | 14 (12) | 2 (2) | 4 (16) | 1 (4) | 6 (19) | - | 2 (11) | - | 5 (8) | 1 (2) | 3 (13) | 1 (4) | 2 (14) | 1 (7) | 18 (13) | 3 (2) |
| Appointment length | 9 (8) | - | 2 (8) | - | 1 (3) | - | 5 (28) | - | 1 (2) | - | - | - | 4 (29) | - | 11 (8) | - |
| Differences in turnaround time | 25 (21) | - | 5 (20) | - | 11 (35) | - | 5 (28) | - | 10 (17) | - | 5 (22) | - | 1 (7) | - | 30 (21) | - |
| Differences in test quality | 32 (27) | - | 5 (20) | - | 12 (39) | - | 4 (22) | - | 11 (19) | - | 8 (35) | - | 3 (21) | - | 37 (26) | - |
| Personal interest in disorder | - | - | 2 (8) | - | 1 (3) | - | - | - | 1 (2) | - | - | - | - | - | 2 (1) | - |
| Frustration with lack of funding | 9 (8) | 2 (2) | 4 (16) | - | 4 (13) | - | 2 (11) | 1 (6) | 3 (5) | 1 (2) | 2 (9) | - | 2 (14) | - | 13 (9) | 2 (1) |
| Disagreement with funding decisions | 19 (16) | 1 (1) | 7 (28) | 1 (4) | 9 (29) | 2 (6) | 5 (28) | - | 10 (17) | - | 1 (4) | - | 1 (7) | - | 26 (18) | 2 (1) |
| Impact on medical management | 76 (64) | 27 (23) | 18 (72) | 10 (40) | 25 (81) | 8 (26) | 12 (67) | 4 (22) | 35 (59) | 16 (27) | 17 (74) | 7 (30) | 6 (43) | 3 (21) | 94 (65) | 37 (26) |
| Impact on family planning | 80 (67) | 6 (5) | 17 (68) | 6 (24) | 23 (74) | - | 12 (67) | - | 40 (68) | 3 (5) | 14 (61) | 1 (4) | 9 (64) | 2 (14) | 97 (67) | 6 (4) |
| Psychological impact of having results | 60 (50) | - | 10 (40) | - | 20 (65) | - | 10 (56) | - | 21 (36) | - | 12 (52) | - | 6 (43) | - | 70 (49) | - |
| Other | 9 (8) | 5 (4) | 2 (8) | - | 2 (6) | 1 (3) | 3 (17) | 1 (6) | 5 (8) | 3 (5) | - | - | - | - | 11 (8) | 5 (3) |

^a^ Category totals may be discordant due to “check all that apply” questions; percentages are calculated as percent of participants rather than percent of total responses.

^b^ An open-response field allowed participants who selected “other” to describe those circumstances; responses included: the patient asking about other/private pay options, reflexing to a larger but non-funded panel if the funded test result is uninformative, if the results were necessary for the lab’s development of a subsequent test, wait time to be seen in the clinic, and working in a private clinic.
