# Supplementary material for: Canadian genetic healthcare professionals’ attitudes towards discussing private pay options with patients
Source: Mol Genet Genomic Med. 2019 Feb 2;7(4):e00572. doi: 10.1002/mgg3.572 (PMC6465662; doi:10.1002/mgg3.572)
Supplement: Supplementary file 5 [file MGG3-7-na-s005.docx]

**Supplementary Table 5:** Views on whether the discussion of private pay options is ethical and which ethical principles most influence that view.

|  | GC (%)  n=119 | MD (%)  n=25 | Cancer (%)  n=69 | Prenatal (%)  n=62 | Other (%)  n=110 | BC (%)  n=31 | Prairies (%)  n=18 | ON (%)  n=59 | QC (%)  n=23 | Maritimes (%)  n=14 | Total (%)  n=144 |
| --- | --- | --- | --- | --- | --- | --- | --- | --- | --- | --- | --- |
| Is discussing private pay ethical? | | | | | | | | | | | |
| Yes | 78 (66) | 16 (64) | 40 (58) | 36 (58) | 75 (68) | 24 (77) | 10 (56) | 37 (63) | 16 (70) | 8 (57) | 94 (65) |
| No | 1 (1) | - | 1 (1) | 1 (2) | 1 (1) | - | - | 1 (2) | - | - | 1 (1) |
| Sometimes | 40 (34) | 9 (36) | 28 (41) | 25 (40) | 34 (31) | 7 (23) | 8 (44) | 21 (36) | 7 (30) | 6 (43) | 49 (34) |
| Which bioethical principle most significantly influenced your answer to the question above? | | | | | | | | | | | |
| Justice | 14 (12) | 3 (12) | 11 (16) | 6 (10) | 13 (12) | 4 (13) | 3 (17) | 4 (7) | 4 (17) | 2 (14) | 17 (12) |
| Autonomy | 66 (55) | 14 (56) | 38 (55) | 35 (56) | 61 (55) | 17 (55) | 9 (50) | 31 (53) | 12 (52) | 10 (71) | 80 (56) |
| Non-maleficence | 8 (7) | 2 (8) | 3 (4) | 5 (8) | 9 (8) | 3 (10) | 1 (6) | 4 (7) | 2 (9) | - | 10 (7) |
| Beneficence | 31 (26) | 6 (24) | 17 (25) | 16 (26) | 27 (25) | 7 (23) | 5 (28) | 20 (34) | 5 (22) | 2 (14) | 37 (26) |
