# Supplementary material for: Canadian genetic healthcare professionals’ attitudes towards discussing private pay options with patients
Source: Mol Genet Genomic Med. 2019 Feb 2;7(4):e00572. doi: 10.1002/mgg3.572 (PMC6465662; doi:10.1002/mgg3.572)
Supplement: Supplementary file 6 [file MGG3-7-na-s006.docx]

**Supplementary Table 6:** Geographic distribution of full members of the Canadian Association of Genetic Counsellors (CAGC) compared to survey participants.

|  | CAGC Full membership* (%) n=291 | Survey Participants (%) n=144 |
| --- | --- | --- |
| British Columbia | 56 (19) | 28 (24) |
| Alberta, Saskatchewan, & Manitoba | 39 (13) | 13 (11) |
| Ontario | 127 (44) | 50 (41) |
| Quebec | 47 (16) | 21 (18) |
| Newfoundland/Labrador, Nova Scotia, New Brunswick, & PEI | 21 (7) | 16 (13) |
| Territories | 1 (0) | 1 (1) |

*Geographic distribution of full CAGC members was received through personal communication with the CAGC office. This table only includes members located in Canada; 20 international members were excluded from the calculations.
