# Supplementary material for: Canadian genetic healthcare professionals’ attitudes towards discussing private pay options with patients
Source: Mol Genet Genomic Med. 2019 Feb 2;7(4):e00572. doi: 10.1002/mgg3.572 (PMC6465662; doi:10.1002/mgg3.572)
Supplement: Supplementary file 7 [file MGG3-7-na-s007.docx]

**Supplementary Material: Questionnaire**

Please Note: “Private Pay” refers to any clinically indicated tests paid for by any entity other than the provincial government (e.g. self-pay). It does not refer to direct-to-consumer testing.

1. What is your designation? (Check all that apply)
   - Clinical Geneticist (MD)
   - Genetic Counsellor
   - PhD Geneticist
   - Nurse
   - Other: _______________________
2. What is your gender?

- Male
- Female
- Other
- Prefer not to specify

1. In what language(s) do you primarily practice?

- English
- French
- Both English and French

1. For how many years have you been practicing in genetics/genetic counselling?

- 0-2 years
- 3-5 years
- 6-10 years
- 11-20 years
- >20 years
- I am not currently practicing (seeing patients)

1. In which province/territory do you currently practice? (Check all that apply)
   - Alberta
   - British Columbia
   - Manitoba
   - New Brunswick
   - Newfoundland and Labrador
   - Northwest Territories
   - Nova Scotia
   - Nunavut
   - Ontario
   - Prince Edward Island
   - Quebec
   - Saskatchewan
   - Yukon
   - I am not currently practicing (seeing patients) in Canada
2. In what area(s) of genetics do you primarily practice? (Check all that apply)
   - Adult
   - Cancer
   - Cardiac
   - Laboratory
   - Metabolic
   - Pediatric
   - Prenatal
   - Psychiatric
   - Other: _______________________
3. In what type of institution do you practice? (Check all that apply)
   - Academic/university health sciences centre
   - Hospital clinic
   - Private clinic
   - Industry
   - Government agency
   - Other: _______________________
4. Does your clinic/program have a policy regarding the discussion of private pay options for genetic testing with your patients?
   - Yes, the policy prevents or discourages this discussion
   - Yes, the policy promotes or encourages this discussion
   - No
   - I don’t know

8. b) IF YES to #8: Is it a formal policy or an unwritten understanding?

- - Formal, written policy
  - Informal agreement/unwritten understanding

8. c) IF YES to #8: Do you agree with that policy?

- - Yes
  - No

1. Do you ever discuss private pay options for genetic testing with patients?
   - Yes
   - No

9. b) IF YES to #9: How often do these discussions arise?

- - Almost every clinic day
  - Approximately once per week (i.e. once per 5 clinic days)
  - Approximately once per month (i.e. once per 20 clinic days)
  - Less frequently than once per month

1. Under what circumstances would you discuss private pay options for genetic testing with patients? (Check all that apply)
   - Whenever private pay is an option
   - When a test would be informative/beneficial but is not automatically funded by the government and/or a government funding request has been denied
   - Only when I think the patient can afford to pay for the test him/herself
   - Only when a patient brings it up first
   - Only for specific circumstances and/or diagnoses (Please elaborate below:) __________________________________________________________________________________________________________________________________________________________
2. What types of private pay options for genetic testing would you discuss with patients? (Check all that apply)
   - Non-Invasive Prenatal Testing (NIPT)
   - First Trimester Screening (FTS)
   - Single gene sequencing
   - Multi-gene panel sequencing
   - Whole exome sequencing (WES)
   - Whole genome sequencing (WGS)
   - Prenatal Genetic Screening (PGS)/Comprehensive Chromosome Screening (CCS)
   - Pre-implantation Genetic Diagnosis (PGD)
   - PGD, PGS, or CCS - but only if I know they are already considering *in vitro* fertilization (IVF)
   - Other: ________________________________________________________________________
3. Which factors would influence your decision to discuss private pay? (Check all that apply)

- Cost of the test in question
- Your perception of the patient’s income
- Your perception of the patient’s understanding
- Patient’s risk (e.g. age, personal or family history, etc.)
- Patient or family’s anxiety level
- Patient or family falls short of ministry’s criteria for funding
- Your stance on a two-tier healthcare system
- Medicolegal issues
- Length of the appointment
- Differences in turnaround time
- Differences in the quality of available tests (e.g. accuracy)
- Your own interest in the disorder in question
- Your frustration with the lack of available funding
- Your disagreement/dissatisfaction with the ministry’s funding decision(s)
- Possible impact on medical management
- Possible impact on family planning
- Possible psychological impact of having test results
- Other: ________________________________________________________________________

12. b) Which one of the factors in this list would you rank as the most influential in your decision-making?

- Cost of the test in question
- Your perception of the patient’s income
- Your perception of the patient’s understanding
- Patient’s risk (e.g. age, personal or family history, etc.)
- Patient or family’s anxiety level
- Patient or family falls short of ministry’s criteria for funding
- Your stance on a two-tier healthcare system
- Medicolegal issues
- Length of the appointment
- Differences in turnaround time
- Differences in the quality of available tests (e.g. accuracy)
- Your own interest in the disorder in question
- Your frustration with the lack of available funding
- Your disagreement/dissatisfaction with the ministry’s funding decision(s)
- Possible impact on medical management
- Possible impact on family planning
- Possible psychological impact of having test results
- Other: ________________________________________________________________________

1. Do you believe that discussing private pay options for genetic testing with patients is ethical?
   - Yes
   - No
   - Sometimes

13. b) Please rank the following ethical principles in terms of how significantly they weighed in your decision-making above, from most (#1) to least (#4):

_#_ Justice (providing equal access to healthcare and/or avoiding use of government resources to coordinate non-funded testing)

_#_ Autonomy (a patient’s right to know all of his/her options and make a decision for him/herself)

_#_ Non-maleficence (“do no harm”; avoiding potential psychological harm that may result from informing patients about tests/options that they cannot afford)

_#_ Beneficence (presenting non-funded testing that could potentially benefit the patient)

13. c) Please elaborate if you would like (optional): ________________________________________________________________________________________________________________________________________________________________________

1. Do you have any comments about the above questions or this topic in general that you would like to share? ________________________________________________________________________________________________________________________________________________________________________________________________________________________________________________________________________________________________________________________________________________

**Supplementary Table 1:** Geographic distribution of full members of the Canadian Association of Genetic Counsellors (CAGC) compared to survey participants.

|  | CAGC Full membership* (%) n=291 | Survey Participants (%) n=144 |
| --- | --- | --- |
| British Columbia | 56 (19) | 28 (24) |
| Alberta, Saskatchewan, & Manitoba | 39 (13) | 13 (11) |
| Ontario | 127 (44) | 50 (41) |
| Quebec | 47 (16) | 21 (18) |
| Newfoundland/Labrador, Nova Scotia, New Brunswick, & PEI | 21 (7) | 16 (13) |
| Territories | 1 (0) | 1 (1) |

*Geographic distribution of full CAGC members was received through personal communication with the CAGC office. This table only includes members located in Canada; 20 international members were excluded from the calculations.

**Supplementary Table 2:** Types of genetic testing that are discussed as private pay options.

|  | Cancer Only (%) n=23 | All Others (%) n=121 | Total (%)  n=144 |
| --- | --- | --- | --- |
| Which types would you discuss?a | | | |
| Non-invasive prenatal testing (NIPT) | 3 (13) | 86 (71) | 89 (62) |
| First Trimester Screening (FTS) | 1 (4) | 12 (10) | 13 (9) |
| Single gene sequencing | 7 (30) | 53 (44) | 60 (42) |
| Multi-gene panel sequencing | 23 (100) | 82 (68) | 105 (73) |
| Whole exome sequencing | 2 (9) | 46 (38) | 48 (33) |
| Whole genome sequencing | 1 (4) | 14 (12) | 15 (10) |
| Pre-implantation genetic screening (PGS/CCS) | 1 (4) | 35 (29) | 36 (25) |
| Pre-implantation genetic diagnosis (PGD) | 7 (30) | 77 (64) | 84 (58) |
| PGD/PGS/CCS only if already considering in vitro fertilization (IVF) | 4 (17) | 22 (18) | 26 (18) |
| Other^b^ | 1 (4) | 13 (11) | 14 (10) |

^a^ Category totals may be discordant due to “check all that apply” questions; percentages are calculated as percent of participants rather than percent of total responses.

^b^ An open-response field allowed participants who selected “other” to name other types of genetic tests; responses included carrier testing, expanded carrier screening, microarray, pharmacogenetic testing, karyotype, and tumour testing.

**Supplementary Table 3:** Views on whether the discussion of private pay options is ethical and which ethical principles most influence that view.

|  | GC (%)  n=119 | MD (%)  n=25 | Cancer (%)  n=69 | Prenatal (%)  n=62 | Other (%)  n=110 | BC (%)  n=31 | Prairies (%)  n=18 | ON (%)  n=59 | QC (%)  n=23 | Maritimes (%)  n=14 | Total (%)  n=144 |
| --- | --- | --- | --- | --- | --- | --- | --- | --- | --- | --- | --- |
| Is discussing private pay ethical? | | | | | | | | | | | |
| Yes | 78 (66) | 16 (64) | 40 (58) | 36 (58) | 75 (68) | 24 (77) | 10 (56) | 37 (63) | 16 (70) | 8 (57) | 94 (65) |
| No | 1 (1) | - | 1 (1) | 1 (2) | 1 (1) | - | - | 1 (2) | - | - | 1 (1) |
| Sometimes | 40 (34) | 9 (36) | 28 (41) | 25 (40) | 34 (31) | 7 (23) | 8 (44) | 21 (36) | 7 (30) | 6 (43) | 49 (34) |
| Which bioethical principle most significantly influenced your answer to the question above? | | | | | | | | | | | |
| Justice | 14 (12) | 3 (12) | 11 (16) | 6 (10) | 13 (12) | 4 (13) | 3 (17) | 4 (7) | 4 (17) | 2 (14) | 17 (12) |
| Autonomy | 66 (55) | 14 (56) | 38 (55) | 35 (56) | 61 (55) | 17 (55) | 9 (50) | 31 (53) | 12 (52) | 10 (71) | 80 (56) |
| Non-maleficence | 8 (7) | 2 (8) | 3 (4) | 5 (8) | 9 (8) | 3 (10) | 1 (6) | 4 (7) | 2 (9) | - | 10 (7) |
| Beneficence | 31 (26) | 6 (24) | 17 (25) | 16 (26) | 27 (25) | 7 (23) | 5 (28) | 20 (34) | 5 (22) | 2 (14) | 37 (26) |
